# Navigating the AI era in dietetics: a qualitative analysis of professional identity, ethical concerns, and future projections in Türkiye

**DOI:** 10.1186/s12913-026-14995-0

**Published:** 2026-06-30

**Authors:** Kadriye Toprak, Makbule Ercakir, Pinar Gobel

**Affiliations:** 1 Department of Nutrition and Dietetics, School of Health Sciences, Ankara Medipol University, Altındağ, Ankara 06050 Türkiye; 2 Independent Researcher, Çankaya, Ankara 06690 Türkiye; 3 Department of Nutrition and Dietetics Gülhane School of Health Sciences, University of Health Science, Keçiören, Ankara 06010 Türkiye

**Keywords:** Artificial intelligence, Dietetics, Nutrition, Qualitative research, Technology perception, Health technologies

## Abstract

**Background:**

As artificial intelligence (AI) technologies become increasingly integrated into healthcare systems, understanding their impact on healthcare professionals is essential. This study provides a comprehensive examination of dietitians’ perspectives, experiences, and future projections regarding AI in Türkiye. The research elucidates how artificial intelligence may affect professional identity, patient-dietitian interactions, and ethical boundaries, drawing on direct insights from practitioners.

**Methods:**

This qualitative research was conducted with 16 dietitians working in different institutions (hospitals, universities, and counselling centres) in Türkiye, selected through purposive sampling. Data were collected via a semi-structured interview (face-to-face/online) and analysed using inductive thematic analysis.

**Results:**

The analysis indicates that dietitians’ perspectives on artificial intelligence are influenced by both perceived opportunities and risks. Participants described artificial intelligence as a valuable assistant that enhances information retrieval and efficiency yet expressed concerns regarding misinformation and the erosion of professional boundaries. A prevailing view among participants is that artificial intelligence can perform technical calculations but cannot replicate essential human attributes in dietetics, such as empathy, emotional connection, and personalized care. Furthermore, participants highlighted that information pollution generated by artificial intelligence may undermine client trust, with data privacy identified as the most significant ethical concern.

**Conclusions:**

Dietitians maintain a cautious yet optimistic perspective regarding artificial intelligence technologies. Participants generally do not view technology as a competitor that will replace dietitians, but rather as a complementary tool that supports guidance and coaching beyond information provision. To maximize the benefits of artificial intelligence and mitigate associated risks, revising educational curricula to include digital literacy and promptly establishing professional ethical standards are essential.

**Supplementary Information:**

The online version contains supplementary material available at 10.1186/s12913-026-14995-0.

## Background

Artificial Intelligence (AI) encompasses computer systems capable of mimicking human cognitive processes, executing complex problem-solving tasks, recognizing high-dimensional patterns, and engaging in autonomous decision-making [1–3]. Together with its subfields, such as Machine Learning (ML) and Deep Learning (DL), AI has emerged as a transformative force in healthcare, serving as the foundation for data-driven interventions. Its capacity to analyze vast and complex datasets, identify intricate biological patterns, and generate personalized recommendations is expected to revolutionize the field of nutrition and dietetics [1, 4].

Applications of AI in nutrition science span a broad spectrum, ranging from individual dietary assessment to the management of chronic diseases. AI-based systems hold significant promise for rendering traditional dietary assessment processes more accurate, efficient, and objective [4]. Through advanced image recognition and portion size estimation capabilities, the energy and macronutrient content of a meal can be determined with high precision solely from smartphone photographs [5, 6]. By providing personalized nutritional advice that helps individuals understand their eating habits, these technologies are effectively digitizing the Nutrition Care Process (NCP) [7, 8].

Furthermore, AI plays a critical role in the prediction, diagnosis, and management of nutrition-related chronic conditions, including obesity, type 2 diabetes, and other metabolic disorders [7, 8]. The “precision nutrition” paradigm which integrates genetics, the microbiome, and real-time metabolic data (such as CGM) enables the development of interventions tailored to an individual’s unique response to nutrients [7, 8]. Evidence suggests that such applications offer a strategic economic solution for employers and health systems by reducing healthcare costs [4, 8]. Additionally, “Digital Twin” technology, which simulates an individual’s biological data, demonstrates revolutionary potential in predicting disease remission. Current research focuses not only on treatment but also on preventive health goals by supporting weight maintenance in normal-weight individuals [8].

However, the integration of AI into clinical practice presents substantial challenges regarding professional roles, ethics, and practical implementation [9, 10]. Dietitians have expressed skepticism regarding the reliability and accuracy of advice provided by generative AI tools like ChatGPT, which have demonstrated deficiencies in fundamental areas such as interpreting anthropometric measurements and monitoring the nutrition care process [6, 11]. This situation creates a “professional identity threat,” where experts feel their professional standing is at risk and the value of their expertise is being diminished [12, 13]. Moreover, ethical concerns—including bias in training data, data privacy issues, and a lack of transparency (the “black box” phenomenon)—may cause users to approach AI-generated information with caution [9, 10].

While the current literature predominantly addresses the technical applications and potential of AI [1–3, 14], there is a notable paucity of research examining the direct impact of AI on practitioners and their perceptions. Recent findings from the United States by Liu et al. [14] indicate that while 58% of Registered Dietitian Nutritionists report some familiarity with AI, only 13% consider themselves very or extremely familiar, highlighting a significant competency gap among experts [14]. This qualitative study aims to bridge this gap by comprehensively analyzing dietitians’ perceptions, experiences, professional concerns, and ethical considerations regarding AI. The findings intend to shed light on the responsible and effective integration of AI into clinical practice.

## Methods

### Research design

In this study, a qualitative research design was adopted to deeply understand the underlying reasons and context of this phenomenon, rather than superficially measuring dietitians’ perceptions, experiences, and views of AI. This design offers an ideal approach to reveal how participants interpret the world from their own perspectives. To ensure methodological rigor, transparency, and comprehensive standards in data reporting, the study design and reporting process adhered to the Consolidated Criteria for Reporting Qualitative Research (COREQ) guidelines [15].

### Participants and sample

The study population consists of dietitians actively working in Türkiye. Participants were selected using purposive sampling, a non-probability sampling technique. Purposive sampling was chosen to focus on ‘information-rich’ cases that could provide comprehensive and detailed data relevant to the research problem [16]. A maximum variation sampling strategy was employed to ensure diversity and breadth of perspectives.

The sample size was determined according to the principle of data saturation, a common methodology in qualitative research [17]. Data saturation is the point at which no new themes or significant information emerge from additional interviews, and the aim was to reach this threshold with approximately 15–25 participants. The interviews revealed that no new concepts or themes emerged after the 16th participant. Data collection and analysis were conducted concurrently to monitor saturation. In this study, saturation was conceptualized as informational and thematic redundancy, defined as the point at which no new open codes emerged from successive interviews and no additional information contributed to the refinement of existing themes or sub-themes. Through continuous thematic tracking, the research team observed that the final interviews generated repetitive information and did not produce novel code variations or modifications to the thematic structure. Consequently, conceptual saturation was rigorously achieved at both the granular code and broader sub-theme levels by the sixteenth interview, confirming that the sample size (*n* = 16) was robust enough to fully address the research questions. To be included in the study, participants were required to hold a bachelor’s degree in nutrition and Dietetics, have at least one year of work experience, and volunteer to participate in the study. Those who were not actively working as dietitians and those with difficulties that would prevent communication were excluded from the study.

### Ethical considerations

Ethical committee approval for this study was obtained from the Ankara Medipol University Non-Interventional Clinical Research Ethics Committee on 02.12.2025 and numbered 221. Potential participants were provided with detailed information about the purpose of the research, how the data would be used, and how confidentiality would be ensured. An ‘Informed Consent Form’ was obtained from all participants. Interview recordings and transcripts were anonymised (coded as P1, P2, etc.) and stored securely.

### Data collection tools

Data were collected using a semi-structured interview form developed specifically for this study by the researchers in line with the relevant academic literature [18–21] and the objectives of the study. This form was chosen to ensure that all participants were asked the same basic questions (structured part) while also allowing for the flexible exploration of participants’ individual experiences and unexpected views (unstructured part). In addition to demographic information, the interview form contained eight open-ended main questions (AI perception, experience, advantages/challenges, patient relationship, ethics, future vision, etc.). The questions have been revised and finalized based on feedback from field experts regarding their content validity and suitability for qualitative research. The English version of the interview questions is included in Supplementary File 1.

### Data collection process

Data collection occurred in December 2025. Participants were recruited through professional associations, social media groups, and the researchers’ professional networks. An announcement describing the study’s aim, eligibility criteria, and voluntary nature was shared with professional dietetics association mailing lists and relevant social media groups. In addition, to ensure diversity in terms of institution type and area of practice, some participants meeting the eligibility criteria were directly identified and invited by the research team from within their professional networks. To minimize potential self-selection bias whereby only technology-enthusiastic dietitians might have volunteered, the announcement explicitly stressed that prior AI experience or technical background was not required, which helped to encourage a broad spectrum of professional views. Furthermore, to address potential social desirability bias during data collection, the researchers maintained a non-judgmental stance during interviews, explicitly reassuring participants that there were no correct or incorrect answers.

Interviews were conducted either in person or via online meeting platforms (Zoom, Microsoft Teams, etc.), depending on participants’ preferences and locations. All interviews were audio-recorded with participants’ consent and lasted an average of 30–60 min. Data collection continued until the research team determined that data saturation had been reached.

### Data analysis

In this study, data obtained from semi-structured interviews conducted to reveal dietitians’ views on the integration of AI into the profession and its impact on professional practices were analysed using inductive thematic analysis [22, 23]. Data analysis was conducted following a systematic process that included data reduction, coding, thematisation, and interpretation, as frequently recommended in qualitative research [24, 25].

The interviews were collected through audio recordings and notes, then transcribed verbatim. To ensure the accuracy of the data, the transcriptions were reviewed again by the researcher. Each participant was numbered P1, P2, P3, etc., to ensure anonymity.

In the first stage, the obtained texts were read repeatedly and divided into units of meaning, and open codes were created based directly on the participants’ statements. During the coding process, the natural structure of the data was maintained, and the researcher’s interpretations were kept to a minimum. Subsequently, codes with similar meanings were grouped together to form sub-themes. An overview of the resulting themes and sub-themes was presented in tabular form to enhance transparency and provide a clear representation of the thematic structure.

To increase the reliability of the coding and thematisation process, 20% of the codes were independently reviewed by a second qualitative researcher, and the inter-coder agreement rate was found to be above 90%. This rate was calculated as a simple percentage agreement using the Miles and Huberman formula [Agreement/(Agreement+Disagreement)x100]. The remaining discrepancies (less than 10%) were resolved through a structured consensus-building process. The researchers held face-to-face meetings to re-examine the raw participant quotations and the context of the disputed codes. Each coder presented their conceptual rationale, and definitions of the sub-themes were iteratively refined until a 100% consensus was reached on the final code assignment [22].

In the final stage, relationships between themes were interpreted, and findings were substantiated with direct quotations from participant statements. Data were reported holistically, combining thematic interpretation with illustrative participant quotations to preserve both analytical transparency and contextual depth. The analysis process adhered to approaches that emphasise transparency and systematic rigor in qualitative data analysis [26, 27].

### Validity and reliability of the research

To enhance the scientific quality and robustness of this qualitative study, the ‘reliability’ framework proposed by Lincoln and Guba [27] was used as a basis. In this context, the following steps were examined:

- Credibility: To ensure that the findings accurately reflected participants’ realities, researcher variation and participant confirmation procedures were implemented.
  - Researcher Variation: The data were coded independently by two researchers, and consensus was reached on the themes.
  - Participant Verification: Preliminary themes and interpretations generated from the analysis were shared with selected participants to verify their accuracy in reflecting participants’ views.
- Transferability: To ensure that the results are meaningful for similar groups, the participant profile and research environment were described in detail.
- Consistency and Verifiability:
  - Expert Review: The analysis process and findings were shared with an expert experienced in qualitative research methodology outside the research team to check the consistency of the analysis and whether the interpretations were data-driven.
  - Researcher’s Positionality and Reflectivity: In qualitative inquiry, documenting the researchers’ professional background is an essential element of reflexivity, as the researcher functions as an instrument of data collection and analysis. The research team consists of three researchers, including university-based academic dietitians and a clinical dietitian holding a PhD degree. The researchers acknowledged that their professional backgrounds and familiarity with current dietetic practices could potentially influence data interpretation. Throughout the analysis process, researchers noted their own assumptions and personal views on AI (by keeping a reflexive journal) in an effort to ensure that interpretations remained data-driven and entirely grounded in the participants’ voices rather than the researchers’ professional preconceptions.

### Declaration of AI and AI-assisted technologies in the writing process

During the preparation of this work, the authors used ChatGPT (OpenAI) specifically for translation and language editing purposes to ensure the clarity of the manuscript. Following the use of this tool, the authors reviewed and edited the translated content to ensure its academic and technical accuracy. To ensure full transparency, it is declared that no AI tools were used during the data coding or thematic analysis stages of this research, and the analysis process was conducted entirely by human researchers. The authors take full responsibility for the final content of the publication.

## Results

Table 1 presents the general characteristics of the participants. The participants’ average age was 31.9 ± 5.37, and their average professional experience was 7.9 ± 5.02 years. Women constituted 62.5% (*n* = 10) of the study group, while men constituted 37.5% (*n* = 6). When examining the participants’ educational levels, it was observed that half of the group (50.0%) held bachelor’s degrees, while the other half held postgraduate specialisation degrees. To enrich the scope of the research, diversity in work areas was considered; participants worked in various specialised fields, primarily clinical, outpatient, and kitchen services (18.8% each), as well as clinical nutrition, endocrinology, bariatric surgery, sports nutrition, and academia.

**Table 1:** General characteristics of the participating dietitians (*n* = 16)

Variables		
**Gender (n %)**	Male	6 (37.5)
	Female	10 (62.5)
**Age (** ${\bf{\bar X}}$ **±SS)**		31.9 ± 5.37
**Marital status (n %)**	Married	10 (62.5)
	Single	6 (37.5)
**Educational status (n %)**	Bachelor’s degree	8 (50.0)
	Master’s degree	7 (43.8)
	Doctorate	1 (6.2)
**Type of institution (n %)**	Public	11 (68.8)
	Private	4 (25.0)
	Individual counselling	1 (6.2)
**Field (n %)**	Clinical	3 (18.8)
	Polyclinic	3 (18.8)
	Clinical nutrition	2 (12.5)
	Endocrinology	2 (12.5)
	Bariatric Surgery	1 (6.2)
	Sports nutrition	1 (6.2)
	Catering services	3 (18.8)
	Academic	1 (6.2)
**Professional experience (year) (** ${\bf{\bar X}}$ **±SD)**		7.9 ± 5.02

Data are presented as mean±standard deviation (${\bf{\bar X}}$*±SD*) for continuous variables and as frequency (*n*) and percentage (%) for categorical variables

The thematic analysis resulted in eight overarching themes, each comprising specific sub-themes that address different aspects of AI integration in dietetics. An overview of this thematic structure is presented in Table 2.

**Table 2:** Overview of the main themes and sub-themes identified through inductive thematic analysis of dietitians’ perspectives on AI

Main Themes (Interview Topics)	Sub-themes
First Impressions and Professional Evaluation of AI	• AI’s First Impressions • Contribution to the Profession and Benefits • Threat and Risk Perception • Usage Limitations and Trust Issues
AI Usage Experience in Professional Practice	• Usage Status and Scope • Experiential Evaluation • Purpose of Use
Potential Benefits, Challenges, and Autonomy	• Benefits Provided by AI • Challenges AI May Pose • Professional Autonomy, Ethics and Reliability
Impact on Communication and Trust Relationship	• Communication and Interaction Dimension • Effects on the Relationship of Trust • Seeking Harmony and Balance
New Skills and Training Requirements	• New Skills and Competencies • Curriculum and In-Service Training Requirements • Lack of Awareness and Uncertainty
Ethical, Legal, and Data Security Issues	• Ethical Sensitivity and Professional Responsibility • Data Security and Privacy Concerns • Accuracy, Transparency and Source Reliability • Legal Regulation and Oversight Requirements • Awareness and Individual Attitudes
Future Projections for the Profession (5–10 Years)	• The View that AI Will Be a Tool that Empowers Dietitians • Concern that AI Could Replace Dietitians • The Indispensability of Human Interaction and Empathy • Necessity of Preserving Professional Identity and Ethical Stance • Uncertainties Related to the Development of AI
Strategies for Successful Integration of AI	• Education and Curriculum Reforms • Role of Professional Organisations and Policy Regulations • Professional Competence and Personal Development • Technological Infrastructure and Access Regulations

AI: Artificial Intelligence. Main themes correspond to the structured interview topics, while constituent sub-themes were identified through systematic thematic analysis of the participant responses

Data obtained from participant views, based on their responses to the question, *‘What first comes to mind when you hear “artificial intelligence”? How do you evaluate it in terms of your profession?’*, revealed differences in perceptions of AI and professional evaluations. Four main sub-themes were identified as a result of the thematic analysis. Overall, the findings reveal that participants perceive AI as both an innovation and an opportunity, as well as a source of uncertainty and threat. While acknowledging the advantages of speed and accessibility offered by the technology, participants also expressed concerns about the lack of professional oversight and ethical control.

Participants in the sub-theme of AI’s First Impressions generally defined AI as ChatGPT, technology, rapid information flow, and a tool that makes life easier. While a large proportion of participants directly equated AI with ChatGPT, some viewed it as a symbol of technological progress. For example, one participant stated, ‘*ChatGPT is coming*,* not in the short term but in the long term*,* I think it will be a tool that will leave us unemployed.*’ (P1), viewing AI as a tool for future transformation. However, some participants defined AI as a general technological phenomenon: ‘*When I hear artificial intelligence*,* the first thing that comes to mind is technology; I use it from time to time*,* and I think it’s useful.*’ (P2). Another participant emphasised the speed of access to information: ‘*When I say artificial intelligence*,* what comes to mind is all the data on the internet network being transferred to you in a second.*’ (P3). These initial impressions demonstrate that, from the very beginning, participants perceived AI not merely as a technology, but as a direct and serious threat to their professional future.

Participants in the sub-theme of Contribution to the Profession and Benefits stated that AI could support their profession, speed up processes, and facilitate access to information. In these views, AI is seen as a tool that complements human labour rather than replacing it. One participant highlighted the contribution of AI in terms of speed and efficiency, stating, ‘*I think artificial intelligence can be used because it provides the answers we seek in our professional work in a matter of seconds.*’ (P3). Similarly, P4 expressed a similar view, stating, ‘*It makes me think of rapid production; it can speed up processes and simplify calculations.*’ Some participants emphasised that this contribution depends on correct usage conditions: ‘*I think it’s useful when used correctly*’ (P8). P15, taking a forward-looking perspective, stated, ‘*Artificial intelligence is a huge invention*,* highly usable in terms of nutrition*,* but it’s still in its infancy.*’

Participants in the Threat and Risk Perception sub-theme viewed AI as a potential negative impact on their profession. Some participants indicated that AI could lead to unemployment, reduce interest in dietitians, or cause the spread of misinformation. P2 expressed a similar concern, stating, ‘*Artificial intelligence could reduce interest in dietitians on social media.*’ Some participants also mentioned control and ethical risks: ‘*A power that I consider unlimited and uncontrollable in the future.*’ (P9) and ‘*Artificial intelligence may not always produce completely accurate results and may lead to wrong conclusions.*’ (P5) underscore concerns about trust and ethics. The concerns regarding accuracy and reliability (Misleading and trustworthiness issues) expressed by the participants here have been coded as a professional risk, as the information provided by AI lacks scientific supervision.

Participants in the sub-theme of Usage Limitations and Trust Issues stated that AI is still in its development phase, its reliability has not been fully established, and some participants are not active users. One participant expressed their indecision, stating, ‘*I don’t see artificial intelligence as very negative*,* but I definitely don’t approach it very positively either.*’ (P13). P15 drew attention to the current limitations of AI, stating, ‘*It is a system that has not yet proven its reliability but could be helpful.*’ P16 emphasised their lack of personal experience, stating, ‘*I am not actively using it in my profession*,* so I am distant from this topic.*’ These views confirm that dietitians do not yet find AI sufficiently reliable and are in a state of cautious waiting regarding its integration into their professional practice.

The participants’ responses to the question, ‘*Have you used AI-based tools in your professional practice before? What was your experience like?*’ revealed differences in the frequency of use, purpose, and level of experience with AI tools. Three main sub-themes were identified from the data obtained. Overall, the findings reveal that the vast majority of participants have experienced AI tools, but the depth of use and level of trust vary.

Participants in the Usage Status and Scope sub-theme stated that they used AI tools with varying frequency and at different levels. Some participants stated that they used tools such as ChatGPT, DeepSeek, or Grok directly. For example, one participant stated, ‘*I used ChatGPT and DeepSeek. It provides accurate information when it is not confused*,* but sometimes it states things we know to be incorrect.*’ (P1), referring to their direct usage experience. Similarly, another participant indicated their purpose of use, stating, ‘*I use Grok; sometimes I ask questions about things I am curious about*,* and I find it useful.*’ (P2). Some participants stated that they only used AI for research or curiosity: ‘*I used artificial intelligence for research but did not use it in practice; I think it needs further development.*’ (P4). In addition, a few participants stated that they had never used it or had only tried it for translation. ‘*I have never used it; I generally use journals.*’ (P8) and ‘*I used it for article translation; it produced a good translation in a short time.*’ (P14) are examples of this situation.

Participants in the Experiential Evaluation sub-theme generally described their experiences with AI tools as positive. Some participants noted that these tools provided speed, ease of access, and practical solutions. One participant emphasised the practical benefit of their experience, stating, ‘*It allowed me to access information instantly while talking to the patient*,* speeding up the process.*’ (P5). Similarly, P7 made a positive assessment, stating, ‘*It is useful in terms of providing instant ideas*,* and it is good because it is personalised.*’ However, some participants stated that their experiences were partially positive but that the systems needed improvement. The statement, ‘*What it offered was insufficient; I think it needs to be further developed.*’ (P4) reflects this view. Additionally, some participants noted that AI is not always reliable. ‘*It provided incorrect information; I asked it first*,* then compared it with a book*,* and there was a significant difference.*’ (P11) drew attention to this issue. These experiences of the participants confirm that the previously mentioned trustworthiness issues can translate into concrete errors in clinical practice.

Participants in the Purpose of Use sub-theme stated that they used AI tools most often to conduct research, access information quickly, or find practical solutions in communicating with patients. One participant emphasised the advantage of accessing information, stating, ‘*Since searching for articles takes time*,* I sometimes ask it quickly; my experience was positive*.’ (P13). Another participant explained how they use it in professional practice: ‘*The patient asks if they should eat pineapple; if they have kidney disease*,* I check immediately*,* and the process speeds up*’ (P5). Some participants also stated that they use these tools for translation and academic content production. *‘I’m going to read an article*,* I need to translate it into English*,* I used it and saw its benefits.’* (P14) clearly illustrates this situation. When examining current usage practices, it is understood that AI has not yet established itself at the center of clinical decision support systems; rather, it remains confined to an ‘assistant’ role that alleviates administrative and cognitive burdens, such as academic research, translation, and rapid information verification.

The participants’ responses to the question, ‘*What are the potential benefits and challenges of artificial intelligence in your profession? What are your thoughts on professional autonomy and reliability?*’ reveal that AI is evaluated in a multidimensional manner in terms of professional benefits, encountered challenges, and ethical-reliability perceptions. Three main sub-themes were identified as a result of the thematic analysis. The findings generally show that participants find AI useful in terms of quick access to information, ease of research, and time savings; however, they are cautious due to the production of false information, loss of professional trust, and ethical risks. In particular, the human-emotional dimension emerged as an indispensable element in preserving professional autonomy.

Participants in the sub-theme of Benefits Provided by Artificial Intelligence stated that AI particularly facilitates access to information, saves time, and simplifies data analysis. One participant emphasised the ease of access to information, stating, ‘*It now brings everything up directly via chat without having to use a browser.*’ (P1). Similarly, P3 drew attention to the time advantage of AI, saying, ‘*It may be possible to access research more quickly.*’ Some participants noted that these tools could be particularly beneficial for individuals with limited access: ‘It’s useful for people who can’t reach a dietitian or leave their homes.’ (P8). P5 explained its contribution in terms of patient interaction with the following words: ‘*Having multiple sources together is a good thing for us.*’

Participants in the sub-theme ‘Challenges Artificial Intelligence May Pose’ stated that the most significant disadvantage of AI is the production of incorrect or incomplete information. Similarly, P9 highlighted the accuracy problem, saying, ‘*It can present a publication with a low level of evidence as high-level information*,* rather than the general consensus.*’ Some participants expressed concern that AI could weaken the professional role. P7 expressed a similar concern, stating, ‘*It may diminish the perception of the profession and replace the dietitian.*’ Participants highlighted the importance of empathy and emotional connection (Importance of empathy), which they regard as the spirit of the dietetics profession, as a central necessity against the mechanical nature of AI: ‘*Because the system is robotic*,* there is no point in looking at the patient’s psychology and making decisions*’ (P1) and ‘*Artificial intelligence cannot understand emotions and may misinterpret them*’ (P8).

Participants in the Professional Autonomy, Ethics and Reliability sub-theme indicated that AI is controversial in terms of professional ethics, reliability and human-centredness principles. Most participants emphasised that the systems still have reliability issues. P3 pointed to uncertainty regarding information reliability, stating, ‘*Is the accuracy of the data I access certain? This is confusing.*’ When evaluated from an ethical perspective, most participants found AI risky. P4 stated, ‘*It is unethical to apply information without verifying whether it is correct.*’ However, some participants indicated that AI could contribute to the profession when used in a controlled and limited manner. ‘*It makes sense not to abandon it entirely*,* but to seek support.*’ (P12) and ‘*It can be used under supervision*,* with the oversight of those who know.*’ (P15) reflect this perspective. These views indicate that AI can only be accepted as an ethical tool through a process of supervision by a dietitian; otherwise, fragmentation issues may transform into a risk of professional error.

The participants’ responses to the question, ‘*How do you think the use of artificial intelligence will affect patient-dietitian communication and the relationship of trust?*’ reveal a strong awareness of the impact of AI on communication quality, mutual trust, and professional interaction. The thematic analysis identified three main sub-themes. The findings generally reveal that participants believe AI cannot replace human-centred interaction and that information conflicts may negatively affect the trust relationship.

Participants in the Communication and Interaction Dimension sub-theme emphasised that the diet process is not limited to creating a nutrition plan, but also requires emotional support, empathy, and one-on-one interaction. In this section, participants deepened the lack of empathy, which they previously referred to as a technical deficiency, into an irreplaceable component of the trust relationship established with the patient. One participant emphasised that AI cannot fulfil the emotional dimension, stating, ‘*People coming and pouring their hearts out is part of the diet process*,* but it is not possible to achieve this through the system.*’ (P1). Similarly, P8 stated, ‘*AI cannot fully understand a person because it cannot understand emotions*,’ while P13 emphasised the indispensability of face-to-face communication, saying, ‘*The ability to empathise and read facial expressions is essential to us.*’ Some participants also expressed concern that the perception that AI could replace dietitians could weaken professional bonds. The statement, ‘*People can create their own diet lists and come in*,* which could negatively affect communication with the dietitian*’ (P7) reflects this situation. Participants in the sub-theme ‘Effects on the Relationship of Trust’ stated that excessive trust in AI could weaken the trust between the dietitian and the patient. In this section, participants substantiated how the loss of professional authority and misinformation issues, previously expressed as a general concern, damage the dietitian-patient trust in clinical practice. P5 expressed that the trust placed in AI could undermine the dietitian’s authority, stating, ‘*Because the patient receives an obvious result*,* they accept it as 100% correct; even if I say something different*,* they may question my knowledge.*’ P9 also shared the same concern, stating, ‘*The patient may feel the need to check what the dietitian has provided*,* which could undermine trust in the process.*’ Some participants emphasised that this situation could lead to conflict and loss of trust in communication. P11’s statement, ‘*Patients ask ChatGPT and come back to me; sometimes they ask questions to test me*,’ shows that AI is changing patient behaviour. However, a small number of participants noted that this effect may not always be negative. ‘*If the patient trusts their dietitian*,* they won’t feel the need to verify with artificial intelligence.*’ (P6) reflects a positive approach to the sustainability of trust through established professional credibility.

Participants in the sub-theme of Seeking Harmony and Balance emphasised the need to strike a balance between AI and human interaction. *‘Dietitians should not shy away from this; people will use it*,* so we need to master it.’* (P4) represents this view. Similarly, P14 emphasised the need for harmony, stating, ‘*Patients now have information at their fingertips*,* so we need to move forward in a different way.*’ P11 also views AI as a guiding tool: ‘*If used correctly*,* artificial intelligence can also positively influence trust.*’ This situation reveals that building trust in the dietitian-patient relationship must now be redefined through transparent technology management and deepening human connections, rather than a hierarchy of information.

The participants’ responses to the question ‘*What new skills will be needed with artificial intelligence? What should be included in the curriculum or in-service training?’* show that they are aware of the technology-based transformation in the dietitian profession, but that this transformation needs to be supported by effective training. Three main sub-themes were identified as a result of the thematic analysis. Overall, the findings reveal that participants believe that, in the age of AI, human and cognitive skills such as communication, ethics, research, and media literacy will become as important as technical skills. In this section, participants defined AI not merely as ‘tool usage,’ but as a new ‘digital dietitian identity’ that blends digital ethics, critical thinking, and media literacy. Participants’ views emphasise the need for a professional development approach that places people at the centre alongside technology for success in the dietitian profession in the age of AI. Participants stated that it is necessary to develop not only the ability to ‘use AI’ but also the ability to understand, direct and control it correctly.

Participants in the New Skills and Competencies sub-theme emphasised that future dietitians must possess not only technical knowledge but also strong communication, critical thinking, and ethical awareness skills. P1 expressed this situation with the statement, ‘*Accessing information is easy*,* but we can make a difference if we can establish two-way communication.*’ Similarly, P14 drew attention to the importance of human interaction in the profession, stating, ‘*Artificial intelligence prepares diet lists*,* but we must establish trust through communication.*’ P8 and P9 highlighted the importance of closely following scientific research in an ever-changing information environment. ‘*How can we access healthy data*,* and what should be the correct search methods in artificial intelligence?*’ (P9) reflects the need for new-generation digital research skills. Furthermore, P15 and P16 emphasised the necessity of professional oversight for guiding AI and evaluating its outcomes, touching upon ethical oversight and information verification responsibilities. These findings demonstrate that in the era of AI, dietetics is evolving from a profession that merely conveys information into a role that verifies the accuracy of information and provides emotional guidance to the patient. The future success of the profession depends on the integration of these human skills, which machines cannot replicate, with technical expertise.

Participants in the Curriculum and In-Service Training Requirements sub-theme pointed to the need for training that would support the controlled and conscious use of artificial intelligence. P7 suggested a profession-based approach, stating, ‘*We can teach courses on how to use artificial intelligence correctly in dietetics.*’ P10 and P11 emphasised the importance of media literacy and digital awareness: *‘There is a method to accessing information; you need to know what you are looking for.’* (P10). P16 highlighted the need for regulation in this area, stating that *‘standards for correct use should be developed’* for both students and academics. These recommendations by the participants reveal that the integration of AI into professional practices is not merely an individual effort, but requires a structural educational process supported by university curricula and ethical standards.

Some participants (P2, P3) in the sub-theme of Lack of Awareness and Uncertainty stated that they did not have sufficient awareness of AI-related skills or that they had not yet formed a clear opinion on the matter. Statements such as ‘*I have no opinion on this matter*’ (P2) and ‘*I am not sure if artificial intelligence can be applied in polyclinics*’ (P3) indicate that there is still cognitive uncertainty among colleagues. These uncertainties among participants show that education is essential for dietitians to actively manage artificial intelligence rather than just observing it from a distance.

The participants’ responses to the question ‘*What ethical and legal issues do you think should be considered in the use of artificial intelligence? Do you have any concerns regarding data security and responsibility?*’ reveal significant uncertainties regarding the ethical, legal, and security dimensions of AI use. Five main sub-themes were identified as a result of the thematic analysis. In general, participants stated that the uncontrolled use of artificial intelligence in the field of health and nutrition could undermine both patient privacy and professional credibility. In this section, while acknowledging that AI is an inevitable technology, participants emphasized the urgent need to fill legal gaps, particularly regarding patient privacy, data storage, and the accuracy of information.

Participants in the sub-theme of Ethical Sensitivity and Professional Responsibility emphasised the need to protect patient confidentiality and observe ethical boundaries when working with AI. P5 expressed this situation with the words, ‘*The patient tells us about their psychological state*,* but if we transfer this to ChatGPT*,* we are telling an artificial intelligence about a memory that is no longer ours.*’ Similarly, P12 drew attention to legal and ethical responsibilities by stating, ‘*Personal data should be under state protection and should not exceed patient privacy.*’ P2, P3, and P7 stated that clearly indicating the source of information is an ethical requirement. P3’s statement, ‘*The source of information from artificial intelligence must be clearly written*,* and the study from which it was taken must be specified*,’ emphasises the principle of scientific accuracy. These data demonstrate that, with the use of AI, participants’ understanding of professional responsibility has expanded beyond merely providing accurate information to include protecting data privacy and ethical security.

Participants in the sub-theme of Data Security and Privacy Concerns stated that the most common concern was the risk of data being stored, shared or misused. P1 summarised this shared concern by stating, ‘*I don’t think anyone is without concerns about data security.*’ P11 explicitly voiced the fear that personal information cannot be protected when using AI: ‘*I shared what I wrote during my thesis process*,* and then it responded to me based on my previous data. At that moment*,* I realised I had shared all my information.*’ P16 took this further, emphasising that data privacy poses a professional risk with the statement, ‘When a data set is presented there, it is a security breach.’ These personal experiences shared by the participants reveal concrete concerns regarding how data is processed in AI systems and confirm that data security is one of the greatest uncertainties in professional practice.

Participants in the sub-theme of Accuracy, Transparency and Source Reliability questioned whether the information provided by AI was based on scientific sources. P9 explained this situation as follows: ‘*Perhaps a security indicator*,* such as a certificate number*,* should be added to the data in artificial intelligence in the future.*’ P10 emphasised the importance of professional oversight, stating, ‘*Dietitians should not directly use AI responses without filtering them through their own knowledge.*’ This sub-theme reveals that the lack of source attribution and transparency in AI systems undermines trust in the healthcare field.

Participants in the sub-theme of Legal Regulation and Oversight Requirements expressed concern that the current system is not regulated from an ethical and legal perspective. P4 stated, ‘*I don’t know if there is an ethical framework yet*,* nor do I know where to turn.*’ while P15 expressed this deficiency by saying, ‘*There is no mechanism to supervise artificial intelligence yet.*’ Some participants indicated that AI tools should be subject to official supervision, certification, or standards in the future. These views demonstrate that for the safe use of AI in the field of health, the establishment of an urgent legal framework is mandatory beyond individual efforts.

Some participants in the sub-theme of Awareness and Individual Attitudes (P6, P13) viewed data security as a general risk in everyday life and therefore stated that they had no particular concerns. P6 trivialised the issue by stating, ‘*Our data is collected everywhere; this is not unique to artificial intelligence.*’ In contrast, P11 and P15 emphasised the need to increase awareness of AI through education and awareness-raising. This situation reveals that the variations in the level of awareness among dietitians directly influence ethical perceptions and security concerns regarding AI.

Participants’ responses to the question, ‘*How do you envision the relationship between the dietitian profession and AI in 5–10 years? Do you think AI could replace dietitians, or would it be a tool that empowers them?*’ reveal two main perspectives on the future of the profession. The view that AI will be a tool that empowers dietitians and concerns about the potential of AI to replace the profession. In addition, participants made noteworthy comments on the indispensability of human interaction, the preservation of professional ethical stance, and the unpredictability of technological changes. The participants’ views were grouped into five main sub-themes. In general, the majority of participants believe that AI will not eliminate the dietitian profession but rather enhance its functionality and speed up processes.

Participants in the sub-theme The View that Artificial Intelligence Will Be a Tool that Empowers Dietitians stated that AI will support the dietetics profession, speed up work processes, and facilitate access to information. P3 summarised this attitude by stating, ‘*I don’t think it will replace us*,* I just think it will empower us.*’ Similarly, P12 and P13 emphasised that the process would strengthen the profession as long as it remains under human control and is managed correctly. This perspective suggests that when AI is integrated into work processes, it serves to accelerate access to information and enhance professional efficiency.

Participants in the sub-theme ‘Concern that Artificial Intelligence Could Replace Dietitians’ stated that the impact of artificial intelligence could increase, particularly in standardisable areas such as weight loss dietetics. P7 indicated that this process has already begun, stating, ‘*It could replace weight loss dietitians because even now*,* people are discarding test results and getting diet lists.*’ P14 went further, stating, ‘Artificial intelligence could replace not only dietitians but also doctors,’ painting a future scenario where the boundaries between professions dissolve. These participant views confirm that technology is perceived as a serious professional threat (professional threat) that can directly replace human labor in standard service areas.

Participants in the sub-theme of The Indispensability of Human Interaction and Empathy emphasized that dietetics is not merely a profession that conveys information but involves establishing emotional bonds. P5 reflected this by stating: ‘*Patients are more convinced when they talk face to face; this is not possible with artificial intelligence.*’ P11 also thought along the same lines, stating, ‘People’s purpose in coming to a dietitian is not only to obtain information but also to be accountable to someone and to be motivated.’ Furthermore, P16 pointed out that AI lack the ability to use facial expressions and convey feelings. These emphases by the participants confirm that despite AI’s ability to provide technical information, ‘human-centered’ elements such as empathy, motivation, and responsibility constitute the irreplaceable core of professional identity.

Participants in the sub-theme of the Necessity of Preserving Professional Identity and Ethical Stance stated that the proliferation of AI will increase the importance of professional responsibility and ethical awareness. P10 stated that ‘*The stance and responsibility of dietitians will be decisive.*’ indicating that differences within the profession will shape this process. P15 found AI to be supportive in information production and preparing educational materials, but emphasised the need to maintain ethical awareness. This situation demonstrates that although AI is a technical assistant, the preservation of professional identity depends on the dietitian’s ability to manage this process as an ethical supervisor.

Participants in the sub-theme Uncertainties Related to the Development of Artificial Intelligence stated that it is difficult to make clear predictions about the future due to the speed of technological progress. P7 expressed this uncertainty by saying, ‘*It has changed so much in just two years*,* I don’t know where it will be in ten years.*’ P1, on the other hand, imagined that technological developments could create specialisation and referral systems, predicting that ‘*Artificial intelligence could refer patients to an endocrinologist or paediatric dietitian.*’ These future projections demonstrate that, rather than perceiving technology as a professional threat, dietitians have the potential to manage it as a strategic tool that enhances specialization and referral mechanisms.

The participants’ responses to the question, ‘*What steps should be prioritised for the successful integration of artificial intelligence into the profession? What should universities and professional organisations do?*’ indicate that education, ethics, professional development, and institutional collaboration are decisive factors in the integration process. The participants’ views were grouped into four main sub-themes. Their views suggest that universities must enhance AI literacy through curriculum updates, while professional organizations should establish standards and ethical guidelines. Ultimately, participants emphasized that successful integration depends not only on technological adoption but also on human-centered professionalism and conscious governance.

Participants in the Education and Curriculum Reforms sub-theme emphasised that, for AI to be successfully integrated, courses in this field must first be offered at universities. P3 clearly expressed this necessity, stating, *‘I want new students to take courses related to artificial intelligence.’* P8, on the other hand, drew attention to the importance of renewal in terms of both content and method, stating, ‘Digital services such as online diets are now part of our lives, so learning correctly is very important.’ Furthermore, P13 and P14 argued that this integration should be carried out in collaboration with computer engineering and the health sector, while P15 suggested that clarifying ethical uncertainties must be a priority in the curriculum. These views confirm that strengthening scientific, technical, and ethical literacy is a foundational requirement for the integration of AI into the profession.

Participants in the sub-theme of the Role of Professional Organisations and Policy Regulations emphasised the necessity of institutional guidance and standardization for professional compatibility. P8 pointed to the pioneering role of professional organisations, stating, ‘*Associations can provide training on this subject for proper guidance.*’ P12 expressed the need for coordination at the national level, stating, ‘*Planning can be done in collaboration with the Ministry.*’ P9 made an innovative suggestion, proposing that a ‘reliable artificial intelligence certification’ system could be established. This sub-theme indicates that the integration process must be supported not only by individuals but also by institutional policies and regulations.

Participants in the Professional Competence and Personal Development sub-theme emphasised that dietitians need to develop both their knowledge base and their technological awareness. P2 pointed to a gap between university theory and professional practice, stating that practitioners need to be better equipped for the field. Emphasizing the importance of working together, P5 noted: ‘*Book knowledge alone is not enough; we can develop it together.*’ These insights suggest that successful integration depends on dietitians’ commitment to continuous learning and professional solidarity.

In the sub-theme of Technological Infrastructure and Access Regulations, recommendations for the controlled and safe use of AI came to the fore. P1 proposed an authorization-based data-sharing model, suggesting that access should be restricted and detailed data should only be available in professional versions. This emphasizes that infrastructure development must be managed from the perspective of data security and competent access policies. All these recommendations demonstrate that the professional future in the era of artificial intelligence will only be possible through the collaborative efforts of academia, professional organizations, and policymakers. A secure, ethics-based, and standardized ecosystem created through this collaboration will guarantee the sustainability of the dietetics profession.

## Discussion

This study employed a qualitative methodology to deeply investigate dietitians’ perceptions of AI, their projections regarding professional roles, and the potential risks and benefits inherent in these technologies. The findings indicate that participants experience a distinct “perception dilemma” regarding AI [28]. While dietitians characterize AI as a unique opportunity for ‘rapid access to information’ and ‘time-saving,’ they simultaneously position it as a significant threat due to concerns over ‘generating misinformation,’ ‘erosion of the professional role,’ and ‘fear of unemployment’ [12, 28]. The observation that concerns such as the threat of unemployment coexist with optimistic assessments indicates that there is no homogeneous attitude among all participants; rather, the views exhibit significant diversity and, at times, contradictions. This dual perception is echoed in recent findings from the United States, where 56% of dietitians view AI’s impact as positive and 70% believe it can improve patient outcomes, despite highlighting significant barriers such as regulatory uncertainty and a lack of validated tools [14]. There are similar studies documenting that while the fear of job loss comes to the forefront for some professionals, others regard technology as an opportunity for professional development [13, 28, 29]. The findings obtained from the current study consistently align with contemporary academic discussions regarding the integration of AI within the discipline of nutrition and dietetics [1, 3, 13, 28, 29].

A predominant concern voiced by dietitians pertains to the clinical reliability of data provided by generative AI tools, such as ChatGPT [3, 30]. Participants’ apprehensions regarding AI’s potential to ‘present low-evidence publications as high-quality information’ (P9) and lead to ‘misdirection’ (P1) closely reflect critical safety concerns discussed in the existing literature [11]. Although generative AI tools demonstrate proficiency in general health advice, independent studies document specific safety vulnerabilities in clinical nutrition tasks that mirror this caution. For instance, Niszczota and Rybicka [31] reported that ChatGPT failed to correctly exclude allergens when generating dietary plans for food allergies, posing life-threatening risks to patients. Similarly, Ponzo et al. [32] evaluated AI recommendations for complex clinical conditions such as sarcopenia and found that the appropriateness rate dropped to 55.5%. Furthermore, a recent study by Bolat Yılmaz et al. [33] on post-bariatric surgery nutrition plans revealed that, while AI was successful in calorie calculations, it produced erroneous plans containing high levels of saturated fat, contrary to clinical guidelines. While these external findings represent empirical evaluations of AI performance, in the context of our qualitative inquiry, they indicate that the participants’ subjective apprehensions are highly consistent with documented clinical risks in the literature [30], reinforcing their view that these tools require careful human oversight.

One of the most consistent themes in the research is the “human factor,” which participants described as irreplaceable by AI [12, 28]. Participants emphasized that ‘face-to-face communication,’ ‘emotional support,’ and ‘empathy’ constitute the ontological foundation of the dietetics profession. The statement by P1, ‘Since the system is robotic, there is no point in making decisions without considering the patient’s psychology,’ echoes a view also expressed in the literature, namely that current AI systems may lack the ‘social skills’ and ‘emotional intelligence’ required for effective counseling [12, 28]. From the participants’ perspective, nutrition is viewed not merely as a biochemical process but as a confluence of complex human factors that AI cannot manage in isolation [3, 30]. In this context, the accounts suggest that the role of the dietitian is perceived as evolving from a mere transmitter of information to that of a “clinical arbiter” who monitors, filters, and interprets AI-generated data for the individual patient [3, 13]. While this perspective is shared by some participants, others define the position of AI within the professional hierarchy as a domain of unresolved uncertainty. However, when evaluating this theme, it is essential to consider that the participants’ emphasis on empathy as a technical deficiency of AI may also stem from an instinct to protect professional identity. Indeed, there is indirect evidence in the literature suggesting that discourses on empathy, emotional support, and clinical judgment function as a symbolic line of defense against job loss and threats to professional identity [34–36]. Therefore, further in-depth studies are needed to understand to what extent dietitians’ strong emphasis on these human competencies is a clinical necessity and to what extent it represents a reflex of resistance to technological change.

Although a majority of participants approach AI with caution, they view it not as a “rival,” but as a “collaborative partner” that alleviates routine workloads [3, 28]. Participants anticipated that by accelerating tasks such as research and complex calculations, AI can afford dietitians the time to focus on the human connection with their clients [1, 4]. The literature supports the potential for such collaboration, particularly in automated dietary assessment [3, 5] and reducing ‘self-reporting’ (recall) biases [1]. Moreover, these technical advantages may offer strategic economic solutions by reducing healthcare costs through precision nutrition applications and “Digital Twin” technologies, which may revolutionize disease remission prediction and support preventive health goals [37, 38].

Finally, participants underscored that elements such as ‘ethical frameworks,’ ‘curriculum updates,’ and ‘institutional guidance’ are critical for successful integration [28]. Participants’ framing of AI not merely as a technical tool but as part of a new “digital dietitian identity” highlights their view that curriculum reform should extend far beyond technical skills. However, it is essential to emphasize that this orientation was not shared by all participants: some (P2, P3) exhibited a distinct lack of awareness regarding what these new AI-related skills might entail and were unable to articulate a clear perspective. The recommendations discussed here, therefore, reflect the perspectives of participants who interact more actively with AI, rather than the entire professional group. Furthermore, data security concerns, exemplified by one participant’s realization that their own academic data was being fed back to them by the system, highlight the need for urgent legal regulations and standardized practice guidelines beyond theoretical ethical debates. Concerns raised regarding data privacy and confidentiality are at the center of ethical debates in the literature [9, 10]. While approaches like “Trade-off Hacking,” which balance algorithmic performance with explainability, offer safe innovation models [39], the most rational pathway to establishing trust is education. The cognitive uncertainties and lack of awareness among some dietitians regarding methods of accessing information suggest that the proposed AI literacy training [7, 40] should be designed not merely as a technical user manual, but as a competency for processing data through a scientific filter [3, 7]. In this regard, participants’ expectations from professional organizations parallel the call for “standardization” frequently highlighted in the literature [4, 10].

The findings from this single-country qualitative study should be interpreted as context-specific insights that may, with caution, resonate with discussions in other settings. Participants’ complex and sometimes contradictory attitudes toward technology, along with their concerns about data accuracy, appear to align with professional concerns observed in similar studies conducted in other countries [14, 28]. Notably, the fear of job loss expressed by some participants echoes themes reported in international samples, although the small and context-specific nature of the current sample does not allow for claims about the structural or universal nature of this concern. For example, the need for ‘digital ethics’ and ‘source verification’ identified in our study is non-generalizable, yet it underscores preliminary questions that could be considered when discussing AI tools in comparable healthcare systems. Similarly, limitations highlighted by participants, such as ‘algorithmic bias’ or ‘misleading information,’ may simply serve as speculative baseline ideas worth documenting for future exploratory studies examining educational curricula, though such applications cannot be inferred directly from the present findings. Therefore, while these findings are grounded in the specific experiences of 16 dietitians in Türkiye and should not be generalized beyond this context, they may nonetheless serve as a preliminary, exploratory reference point for future comparative research. Even with the detailed data provided by this research, there are some limitations. First, the small sample size and the fact that the research was conducted in a single country substantially restrict the transferability and generalizability of the findings to other populations, professional contexts, or healthcare systems. In particular, the emergence of specific themes regarding ethical uncertainties, data privacy, and the call for standardization should be interpreted within the context of Türkiye’s current healthcare landscape, where structured legal regulations and clinical oversight mechanisms for AI technologies are still evolving. Second, since most participants were dietitians working in the public sector, perspectives from different healthcare models might not be fully represented. However, the professional diversity within this study group, spanning distinct fields such as bariatric surgery, endocrinology, clinical nutrition, and catering services, may explain the observed variations in future projections. For instance, apprehensions regarding technological ‘replacement’ were explicitly tied to more standardizable services like weight management (P7, P14), rather than being experienced uniformly across all specialized fields. Additionally, since the study was based on voluntary participation, it may have led to a ‘self-selection bias’ in favor of dietitians interested in technology. Consequently, the captured views may reflect a segment of the profession that is relatively more aware of technological trends, meaning that professional anxieties or resistance toward AI could potentially be more pronounced among practitioners with less technological exposure. Finally, as this research relies on the self-reported views of dietitians rather than direct observation of their clinical practices, the results should be considered within this context.

## Conclusion

The findings of this study and the synthesis of the relevant literature indicate that the dietitians participating in this study held diverse and sometimes divergent perspectives on AI, creating a distinct sense of ambivalence among participants. The participants’ cautious reflections regarding ‘misinformation’ and ‘lack of personalisation’ closely resonate with ongoing concerns in the broader healthcare literature [33]. In the participants’ accounts, while the perceived success of AI in general plans and its functionality in specific technical tasks created a ‘replacement’ concern among some individuals, this concern was counterbalanced by the human-centred competencies discussed above, which participants regarded as central to the profession’s resilience.

It is observed that these two perspectives coexist within this sample, and therefore, no definitive conclusion has been reached regarding which perspective predominates among dietitians more broadly. On the other hand, the fact that some participants perceive AI directly as a ‘threat of unemployment’ (P1, P7) reveals that optimistic expectations regarding technology strengthening the profession were not shared by all participants in this study. The findings obtained in this study do not present a uniform conclusion regarding the role of AI in dietetic practice. While some participants evaluate AI as a supportive tool, another group positions it as a concrete threat to professional autonomy. These findings should be understood as illustrative of the range of perspectives that may exist among dietitians, rather than as representative or generalisable conclusions. When evaluated comparatively with international literature, this diversity may reflect, in an exploratory sense, that the participants’ technology-related views align with broader discussions suggesting that the relationship between the dietetics profession and AI is an ongoing process of negotiation that has not yet been finalized [9, 28]. Future research involving larger and more diverse samples would be needed to determine how this process might be shaped by factors such as ‘educational integration’ [40], the development of ‘ethical standards’ [41], and the supervisory role of dietitians.

In conclusion, based on the self-reported viewpoints of this specific sample, AI appears to be perceived as having potential utility in routine technical tasks; however, these insights cannot be extrapolated to predict broader healthcare system outcomes or definitive professional shifts. Whether these technology-related perceptions will translate into professional autonomy or dependency in the wider profession depends on factors beyond the scope of the current qualitative data. To validate, generalize, or expand upon these exploratory findings, prospective and multicenter research involving larger and more diverse samples is needed.

## Supplementary Information

Below is the link to the electronic supplementary material.

Supplementary Material 1

## Data Availability

The datasets, including anonymized interview transcripts and demographic data, generated and analyzed in this study, are not publicly available to protect participant confidentiality but are available from the corresponding author on reasonable request.

## Abbreviations

AI: Artificial Intelligence
ML: Machine Learning
DL: Deep Learning
NCP: Nutrition Care Process
