# Supplementary material for: Navigating the AI era in dietetics: a qualitative analysis of professional identity, ethical concerns, and future projections in Türkiye
Source: BMC Health Serv Res. 2026 Jun 30;26:1171. doi: 10.1186/s12913-026-14995-0 (PMC13499336; doi:10.1186/s12913-026-14995-0)
Supplement: Supplementary file 1 [file 12913_2026_14995_MOESM1_ESM.docx]

**Semi-Structured Interview Questions**

General perception

1. What is the first thing that comes to mind when you hear the term of artificial intelligence? How do you perceive its role in relation to your profession?

Experience

2. Have you ever used any AI-based tools in your professional practice? Could you describe your experience?

Advantages and Challenges

3. What are the potential benefits and challenges that AI may bring to your profession? What are your thoughts regarding professional autonomy and reliability?

Patient–Dietitian Relationship

4. How do you think the use of AI will affect communication and the relationship of trust between the patient and the dietitian?

Professional Roles, Skills, and Education

5. What new skills will be required with the integration of AI? What topics should be included in the academic curriculum or in-service training programs?

Ethical and Legal Dimensions

6. In your opinion, what are the ethical and legal issues that should be considered in the use of AI? Do you have any concerns regarding data security and professional accountability?

Future Vision

7. How do you envision the relationship between the dietetics profession and AI in the next 5-10 years? Do you believe AI could replace dietitians, or will it serve as a tool that empowers them?

Recommendations

8. Which steps should be prioritized for the successful integration of AI into the profession? What role should universities and professional organizations play in this process?
